# Exploring gene knockout strategies to identify potential drug targets using genome-scale metabolic models

**DOI:** 10.1038/s41598-020-80561-1

**Published:** 2021-01-08

**Authors:** Abhijit Paul, Rajat Anand, Sonali Porey Karmakar, Surender Rawat, Nandadulal Bairagi, Samrat Chatterjee

**Affiliations:** 1 Complex Analysis Group, Translational Health Science and Technology Institute, NCR Biotech Science Cluster, 3rd milestone, Faridabad-Gurgaon Expressway, Faridabad, 121001 India; 2 Centre for Mathematical Biology and Ecology, Department of Mathematics, Jadavpur University, Kolkata, 700032 India

**Keywords:** Computational biology and bioinformatics, Systems biology, Mathematics and computing

## Abstract

Research on new cancer drugs is performed either through gene knockout studies or phenotypic screening of drugs in cancer cell-lines. Both of these approaches are costly and time-consuming. Computational framework, e.g., genome-scale metabolic models (GSMMs), could be a good alternative to find potential drug targets. The present study aims to investigate the applicability of gene knockout strategies to be used as the finding of drug targets using GSMMs. We performed single-gene knockout studies on existing GSMMs of the NCI-60 cell-lines obtained from 9 tissue types. The metabolic genes responsible for the growth of cancerous cells were identified and then ranked based on their cellular growth reduction. The possible growth reduction mechanisms, which matches with the gene knockout results, were described. Gene ranking was used to identify potential drug targets, which reduce the growth rate of cancer cells but not of the normal cells. The gene ranking results were also compared with existing shRNA screening data. The rank-correlation results for most of the cell-lines were not satisfactory for a single-gene knockout, but it played a significant role in deciding the activity of drug against cell proliferation, whereas multiple gene knockout analysis gave better correlation results. We validated our theoretical results experimentally and showed that the drugs mitotane and myxothiazol can inhibit the growth of at least four cell-lines of NCI-60 database.

## Introduction

Chronic progressive diseases such as cancer do not derive from abnormal functioning of a single gene or a single pathway but reflect the disorder of complex intracellular and intercellular networks that link tissues and organs^1^. Various tissues and organs like breast, central nervous system (CNS), colon, lung, ovary, prostate, renal are affected by cancer. Millions of people are suffering from cancer and the number is increasing^2–4^. Available drugs that are used for treating cancer, unfortunately, have many side effects^5^. Therefore, there is an increasing demand for new therapies with better therapeutic windows, implying that the drug will target a particular cell type (such as tumour cells) with no or minimum negative effects on healthy cells. The search for such suitable therapeutic windows is an important and challenging problem in the case of cancer. Another problem with the existing cancer drugs is that a particular drug shows different responses when applied to different individuals. This is because the effects of a drug on a patient depend not only on the interaction with its targets but also on the activities of many other enzymes which form a complex network of metabolic reactions in which the products of a reaction become the substrates of other reactions^6^. This leads to the emerging field of personalized medicine and personalized drug-choice^7^. Thus there is a need for developing new anti-cancer drugs taking care of the above problems and demands for an in-depth mechanistic understanding of cancer^8,9^.

Gene knockout study in cancer cell-lines is used to see the effect of an existing cancer drug or to develop new cancer drug^10–14^. Another approach is the phenotypic screening of drugs in cancer cell-lines to find its effect on the cell growth^10,15–18^. Both of these processes are very costly and time consuming^19–21^. Therefore, a computational method, like metabolic networks, could be a good alternative to find drugs having better selective ability in killing cancerous cells^22–24^. One tool which is particularly suitable to deal with problems like personalized medicine and finding a larger therapeutic window is genome-scale metabolic models (GSMMs)^6^. GSMM is the reconstructions of metabolic networks that connect gene–protein–metabolic reactions^25,26^. Different reactions containing stoichiometric coefficients are used to build a stoichiometric matrix to obtain quantitative information on how different metabolites are linked to each reaction in the network. Through flux balance analysis (FBA) on these GSMMs, one can evaluate the metabolic capabilities of the cell, e.g., the capability of synthesizing biomass building blocks. GSMMs have successfully been used in cancer drug development^27–31^. For example, Folger et al.^27^ made a generic metabolic model of cancer and predicted some growth-supporting genes for cancer, which were validated with experimental shRNA data. GSMMs were also used to predict the putative effects of drugs of DrugBank database^6^, but the results were not compared with the experimental data.

The present study is an attempt to explore gene knockout strategies that apply GSMMs in finding possible targets and mechanisms related to cancer disease. For the analysis, we considered the GSMMs of the NCI-60 cell-lines built by Yizhak et al.^29^ using Personalized Reconstruction of Metabolic models (PRIME) approach. They established their cell-specific model based on molecular and phenotypic data. It is shown that the models can find drug targets that inhibit the proliferation of specific cell-lines. Their models can also infer on the prognosis of breast and lung cancer. In this study, we applied their model for a more comprehensive study on the single and multiple gene knockout effects on the growth rate of the cancer cell-line and compared the results with the online experimental database. We also attempted to capture the underlying mechanisms associated with the observed growth reduction rate due to gene knockout. We further analysed our top-ranked genes to get potential targets which were then validated experimentally. It is also observed that multiple knockout tests give a better correlation with experimental observation than single-gene knockout results.

## Results

### Single gene knockout ranking based on their influence on cancer cell proliferation using the genome-scale metabolic models

We used the study of Yizhak et al.^29^ to obtain the GSMMs of cancer from different tissue types. The networks were made using the molecular (gene expression) and phenotypic data (proliferation rate) of cancer cell-lines by applying PRIME method. These data were used to constrain the bounds on the flux values of the corresponding reactions in metabolic networks. The data for NCI-60 collection were taken from the study by Lee et al.^32^, in which RPMI-1640 was used to grow the cell-lines experimentally. We used 60 cancer metabolic networks across 9 tissue types of NCI-60 panel to find cancer drug targets which inhibit cell growth across all cell-lines. We aimed to rank metabolic genes according to their growth inhibitory effect in cancer cell-lines.

**Figure 1:**
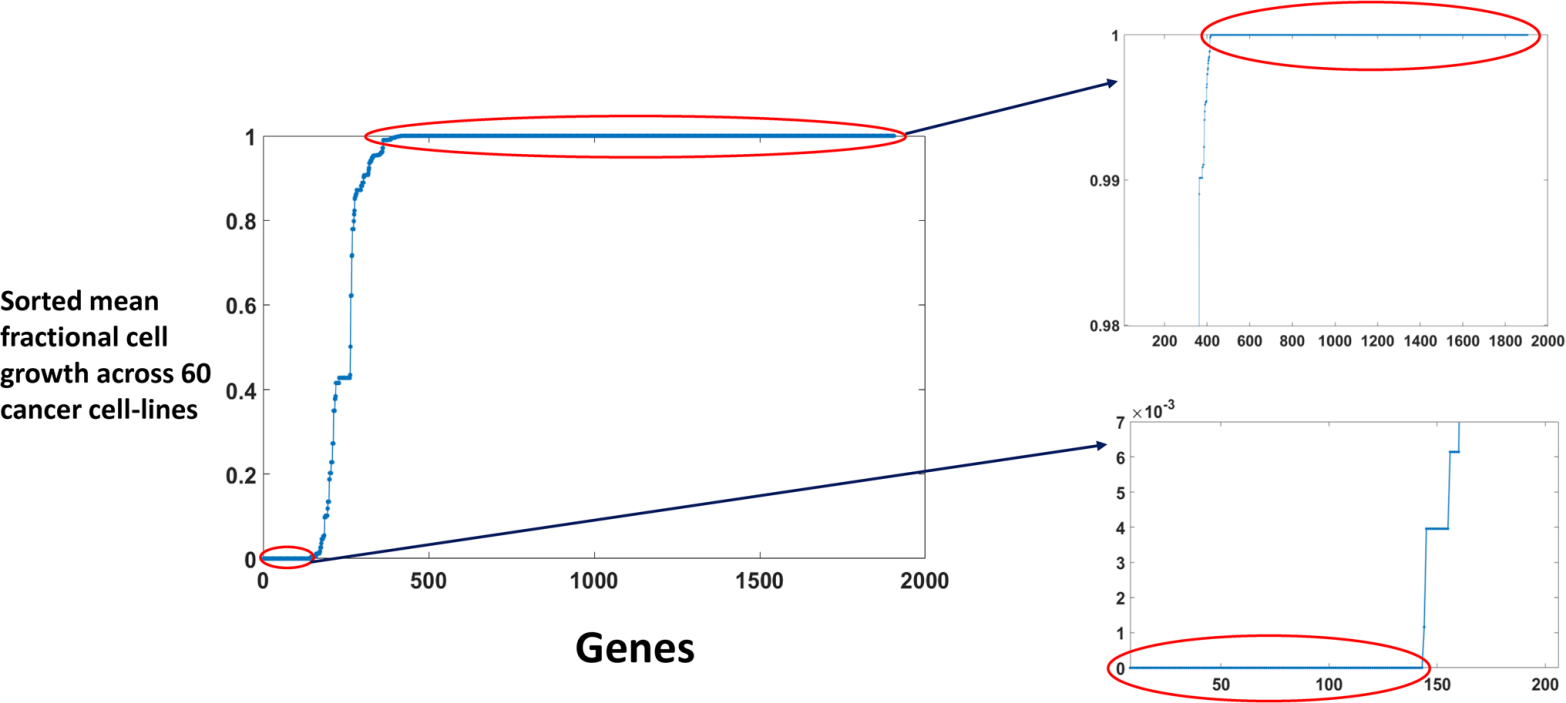
Gene knockout simulation result. Mean value of the fractional cell growth (FCG) across 60 cancer metabolic models for each individual of 1905 metabolic genes. The right lower panel represents the 143 genes which give very low growth rate after knocking out across all 60 cancer models (mean value$<10^{-6}$ & s.d.$<1.2874\times 10^{-6}$) and the upper panel represents the 1488 genes which show no change in the growth rate after knocking out across all 60 cancer models (mean value $>0.99995$ & s.d. $<3.2838\times 10^{-6})$.

Cancer cell-lines metabolic models used in our work contains 1905 genes. We simulated the models via MOMA^33,34^ and predicted the growth rate of cancer cell-lines after knocking out each gene one by one. Taking the average of fractional cell growth (FCG) of 60 cell-lines, we obtained the mean FCG of each 1905 genes (Fig. 1). Using a cut off value of $10^{-6}$ (lower circled portion in Fig. 1) on the sorted mean FCG, we obtained 143 genes that are responsible for very low growth rate in our knockout cancer models (see Supplementary File S1). On the other hand, we got 1488 genes, using a cut off value of 0.99995 (the upper circled portion in the Fig. 1), which show a negligible effect on the growth rate (see Supplementary File S1). Interestingly, looking at the reactions corresponding to the 143 genes, we found that all of them, except one gene, is associated with the coupled reactions (Supplementary Fig. S1).

### Mechanistic insight into the genes giving a low growth rate after knockout

**Figure 2:**
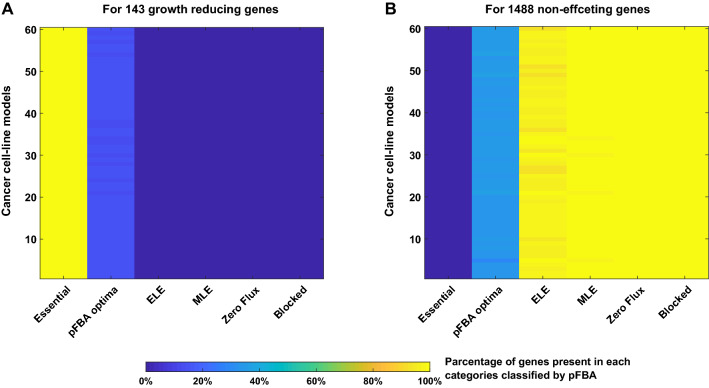
Coverage of pFBA classes by the 143 growth reducing and 1488 non-effecting genes set. For each of the 60 models, pFBA was applied to classify the genes into six categories and then the parentage of involvement for each class into the (**A**) 143 growth reducing genes set and (**B**) 1488 non-effecting genes set was calculated.

We looked for the underlying mechanisms associated with the observed growth reduction rate due to gene knockout. We applied parsimonious enzyme usage FBA (pFBA)^35^ using COBRA Toolbox^36^. pFBA classifies each gene into six categories depending on the optimal growth solutions: essential genes, pFBA optima, enzymatically less efficient (ELE), metabolically less efficient (MLE), zero flux genes and blocked genes. There are 71 essential genes, 470–530 pFBA optima, 230–280 ELE, 545–577 MLE, 82 zero flux genes and 427 blocked genes across 60 cancer cell-lines models. We looked for the classification of the 143 growth reducing genes and found that these genes contain all the 71 essential genes and the rests are pFBA optima (Fig. 2A). On the other hand, all zero flux and blocked genes, with almost all ELE and MLE genes belong to the 1488 non-effecting genes set (Fig. 2B). Though there are some genes from the 1488 set, which are present in pFBA optima class, but no essential genes are there in the 1488 gene set.

**Figure 3:**
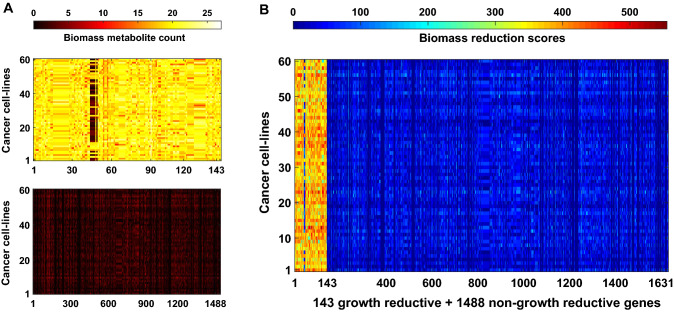
Effect of metabolic genes on biomass function. (**A**) The upper and lower panels indicate the number of biomass metabolites whose production flux at least twofolds decreased by each of the 143 growth reducing genes and 1488 non-growth reducing genes, respectively. For the details of these genes, see Supplementary File S1. (**B**) Biomass reduction scores (BRS) of genes following synergic effect. BRS is high for first 143 growth reducing genes and it is low for 1488 non-growth reducing genes.

The production fluxes of the metabolites involved in the biomass reaction is changed due to gene knockout. The biomass reaction in the GSMMs uses 43 metabolites as substrate, termed as biomass metabolites (see Supplementary Table S1). We measured the fold changes in the production flux of these biomass metabolites under individual gene knockout condition with respect to wild-type condition. The biomass metabolites whose production flux are reduced by more than twofolds are shown in Fig. 3A. The upper panel of this figure shows the number of biomass metabolites associated with 143 genes responsible for growth reduction, and the lower panel shows the number of biomass metabolites associated with 1488 genes, which do not affect the growth rate. One can observe that the number of biomass metabolites associated with the 143 genes is much higher than that of 1488 genes. To confirm the association of 143 genes with the biomass reaction, we introduced a biomass reduction score (BRS) for each gene (see “Method” for details). A gene with higher BRS has more knockout effect on the biomass reaction. It was observed that BRS of 143 genes are much higher than 1488 genes (Fig. 3B), confirming that they are more effective in reducing the flux of biomass reaction.

**Figure 4:**
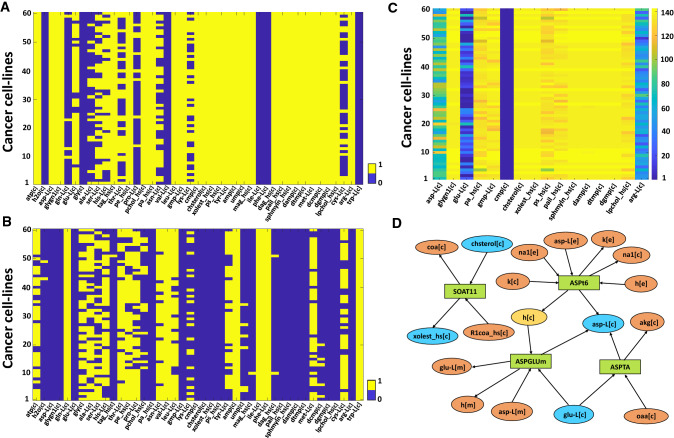
Biomass metabolites affected by the 143 growth reducing genes and 1488 non-effecting genes. Reduction in production fluxes for each individual biomass metabolites were captured in the gene knockout condition. (**A**) Represent the production flux are twofold decreased or not for any 143 genes and (**B**) represents the case for 1488 genes. We gave value 1 if the production flux of a metabolite is decreased in at least one gene knockout condition across each cell-lines, otherwise, the value remains zero. For 16 metabolites, all values are zero across the 60 cancer cell-lines in (**B**) but there are non-zero values in (**A**). (**C**) The number of genes among the set of 143 growth reducing genes whose knockout shows at least twofold reduction in the production fluxes for each of the 16 metabolites. (**D**) A bipartite network that represents the connection between L-Aspartate, L-Glutamate, cholesterol and cholesterol ester. Rectangular and ellipse-shaped boxes indicate the reaction and metabolite respectively and the arrow shows the flow of the flux.

Finally, we looked for the biomass metabolites that are associated specifically with the 143 growth reducing genes. We observed that the production flux of 37 biomass metabolites are reduced by knocking out different genes from 143 genes set (Fig. 4A), while different genes from 1488 genes set reduced production flux of 27 biomass metabolites (Fig. 4B). Calculating the set difference, we obtained 16 biomass metabolites that are specifically associated with different genes from the 143 gene set. It is observed that 12 out of 16 biomass metabolites showed association with most of the 143 genes (see Fig. 4C and Supplementary File S1). Next, we looked for these 16 metabolites whether they are flux coupled or not. If they are flux coupled, then one can expect that the corresponding genes become essential for the production of both for the metabolites. We observed that only 4 metabolites out of 16 are flux coupled (Fig. 4D). L-Aspartate (asp-L[c]) is produced from L-Glutamate (glu-L[c]) by the enzyme kinetic reactions Aspartate Transaminase (ASPTA) but there is another transport reaction L-aspartate transport via Na, H symport and K antiport (ASPt6) in which influx of cytosolic L-Aspartate happens from the extracellular space. For the three cell-lines (SNB-75, HOP-9 and SK-OV-3), flux values of another transport reaction aspartate-glutamate mitochondrial shuttle (ASPGLUm) was observed, which transfer L-Aspartate from mitochondria to cytosol, and as a consequence, cytosolic L-Glutamate enters into the mitochondria. There is another reaction sterol O-acyltransferase (SOAT11) which uses cholesterol (chsterol[c]) to produce cholesterol ester (xolest_hs[c]).

### Finding potential cancer drug targets from the top-ranked genes

Our top-ranked genes can be a potential drug target if their knockout does not significantly affect the growth of non-cancerous cells. So, we need to see the effect of our growth reducing genes on normal cell model. The 60 cell-line panel covers nine different tissues. So, we considered nine models^26^ built on different cell-type from these nine tissues representing their normal condition. The cell-specific models^26^ were built from a global reconstruction (Recon 2), which contains 7440 reactions and 2194 transcripts, using protein expression data from the Human Protein Atlas^37^. These models consist of 2426 ± 467 reactions (± s.d.) and 1262 ± 204 transcripts. They applied a published algorithm^38,39^ to predict the flux activity states of the genes by applying an optimization method. The method maximizes the consistency between gene expression and the corresponding enzyme activity. A comparison of the result of normal cells with that of the cancer cell-lines will help us to find targets that can reduce the growth of cancer cells but has minimal effect on the normal cells. Our gene knockout simulation result gave us 143 genes whose knockout can reduce the growth rate in cancer cell-line metabolic models. Some of these genes have multiple isoforms. After removing those isoforms, the gene list reduced to 121 unique genes and all of them could be potential drug targets. However, to be a potential drug target, a gene should show a minimal activity in normal cells. So, we looked for the activity of these 121 genes on normal cell models and found from the literature^26^ that only 13 genes (Table 1) out of 121 are inactive across all the 9 normal cell models. Thus we have 13 genes that reduce the growth in 60 cancer cell-lines models but have no effect on the flux state of the normal cell models. Therefore, these 13 genes can be considered as potential drug targets against cancer for these 9 tissues with minimal side effects. Interestingly, each of these 13 genes has very high BRS (> 353) (see Table 1). Most of the genes (UQCR11, CYC1, UQCRQ, UQCR10, MT-CYB, UQCRB, UQCRC1, UQCRC2, UQCRFS1 and UQCRH) given in Table 1 have same BRS because they are associated with the same reactions “Ubiquinol-6 Cytochrome C Reductase, Complex III” with *AND* combinations. This reaction is catalysed by cytochrome bc1 complex (EC 1.10.2.2), which is the third complex in the electron transport chain in mitochondria and the subunit proteins are encoded by these ten genes. It plays a critical role in ATP generation process by catalysing electron transfer from ubiquinol to cytochrome c, coupled to proton transport from the matrix space to the intermembrane space of mitochondria^40,41^.

**Table 1:** Gene ID, gene symbol and the corresponding average value of the biomass metabolic score (BRS) across 60 cancer cell-line models of the cancer specific drug targets.

No.	Gene ID	Gene symbol	Avg. of BRS
1	10975	UQCR11	379
2	1537	CYC1	379
3	27089	UQCRQ	379
4	29796	UQCR10	379
5	31	ACACA	385
6	32	ACACB	370
7	4519	MT-CYB	379
8	6646	SOAT1	383
9	7381	UQCRB	379
10	7384	UQCRC1	379
11	7385	UQCRC2	379
12	7386	UQCRFS1	379
13	7388	UQCRH	379

### Experimental validation of the identified potential drug targets

**Figure 5:**
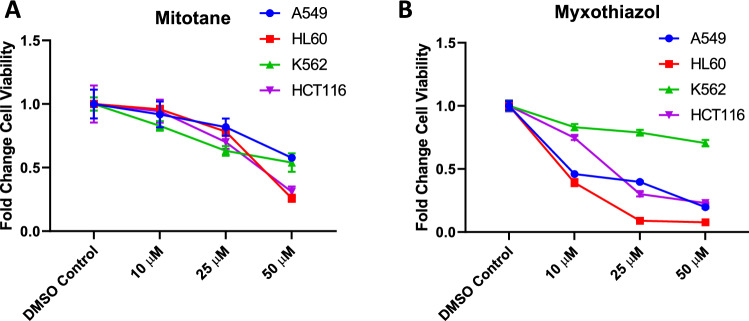
Effect of mitotane and myxothiazol in cell viability. Experimentally measured fold change in cell viability of four different cell-lines (A549, HL60, K562, HCT116) at different concentrations of mitotane and myxothiazol.

To validate our simulation results for identifying potential drug targets, we searched for the inhibitors of these 13 genes and used two commercially available inhibitors, i.e., mitotane (SOAT1 inhibitor)^42^ and myxothiazol (CYTB inhibitor)^43^ for in-vitro studies. Mitotane is reported to show anticancer activity in some cell-lines such as NCI-H295, Hela, HepG2, IMR-32 and HEK293^42^. So we considered four cell-lines (HCT116, K562, HL60 and A549) from NCI-60 cell-line panel, which are different from the cell-lines reported in Ref.^42^. The effect of these two inhibitors on the cell viability was studied by adding the inhibitor at different concentrations and measuring the growth rate of cell-lines. Fold change in the growth rate (i.e., cell viability) was calculated for each cell-line at different drug concentrations for both the drugs and was plotted in Fig. 5. An EC50 value (concentration of drug at 50% fold change cell viability) was calculated for each cell-line from the resultant curve. The EC50 value of mitotane was 37.83 $\upmu$M (for HCT116), 60.85 $\upmu$M (for K562), 38.51 $\upmu$M (for HL60) and 57.99 $\upmu$M (for A549) and for myxothiazol, it was 18.28 $\upmu$M (for HCT116), 84.92 $\upmu$M (for K562), 8.21 $\upmu$M (for HL60) and 9.25 $\upmu$M (for A549). Thus, mitotane and myxothiazol both are effective in inhibiting the growth of these four cell-lines.

### Testing the predictive ability of GSMM for single-gene knockout

**Figure 6:**
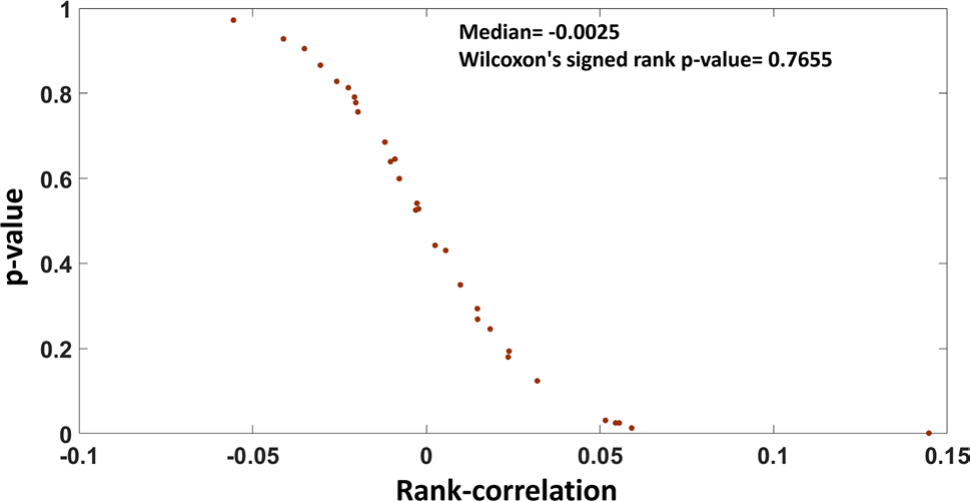
Comparison of gene knockout simulation results with DEMETER database. Distribution of measured Spearman rank-correlation between predicted FCG of genes using GSMM and experimental data from DEMETER for the 30 cell-lines. The *p*-values were measured using permutation test.

We extracted the information from DEMETER database^44^ on the knockdown effect of 1444 genes in 30 cell-lines (Supplementary File S1). We calculated the Spearman rank-correlation between the predicted FCG from our GSMM and the experimental data from DEMETER database for each of the 30 cell-lines. The corresponding *p*-value of the rank-correlation for each cell-line was obtained by permutation test. It was observed that most of the obtained positive rank-correlation were not significant (Fig. 6). There were only 5 cell-lines, which are showing significant positive rank-correlation but their correlation value was less than 0.15. Thus, the obtained gene ranking from single-gene knockout results does not show much correlation with the experimentally observed result. So, we looked for the effect of multiple genes knockout on the growth rate.

### Identification of multiple targets using DrugBank database information

We took the drugs available in the DrugBank database^45^ based on the gene target information. DrugBank database contains the biochemical and pharmacological information about the drugs. We only selected 380 drugs which are inhibitory and the corresponding target genes are present in the cancer cell-lines metabolic models (see Supplementary File S1). These 380 drugs have 202 metabolic targets in the models. To observe the effect of a particular drug on the growth rate, we knocked out all the genes that were inhibited by that drug and simulated the models via MOMA. This exercise was repeated for all the 380 drugs across all the 60 cancer cell-line models. We used a cut off value of 0.5 on FCG (representing at least 50% reduction on the growth rate) to call a drug active (see Supplementary File S1) and obtained 76 drugs. 10 out of these 76 drugs have already been approved in cancer treatment and another 18 drugs are in different phases of the clinical trial.

We considered NCI-60 growth inhibition database^46^ to get the GI50 values of the drugs and compared the predicted anti-proliferative activity with the experimentally measured potency of the drugs. There are 64 drugs with GI50 value in the NCI-60 growth inhibition database. Out of these 64 drugs, 23 drugs have mean log GI50 value less than − 5 across 60 cell-lines and therefore considered to be active against cancer for most of the cell-lines. Comparing these 23 drugs with our list of 76 drugs, we obtained 17 drugs common in both sets.

**Figure 7:**
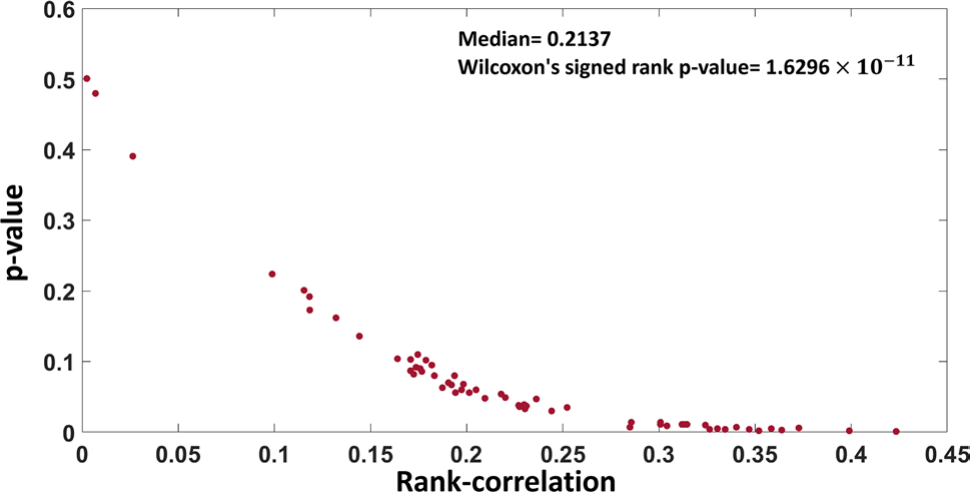
Validation of predicted cell-line specific responses of drugs. Distribution of measured Spearman rank-correlation between predicted FCG of drugs using GSMM and their log GI50 values for the 60 cell-lines. The *p*-values were measured using permutation test.

Finally, a cell-wise comparison between predicted FCG of drugs using GSMM and their log GI50 values was performed and the Spearman rank-correlations was obtained. It is observed that around 50% cell-lines were showing significant rank-correlation and their correlations were also higher than single knockout result, see Fig. 7 (median Spearman rank-correlation = 0.2137, Wilcoxon’s signed-rank *p*-value = $1.6296\times 10^{-11}$).

### Linking the significance of single-gene knockout ranking on the activity of drug

**Figure 8:**
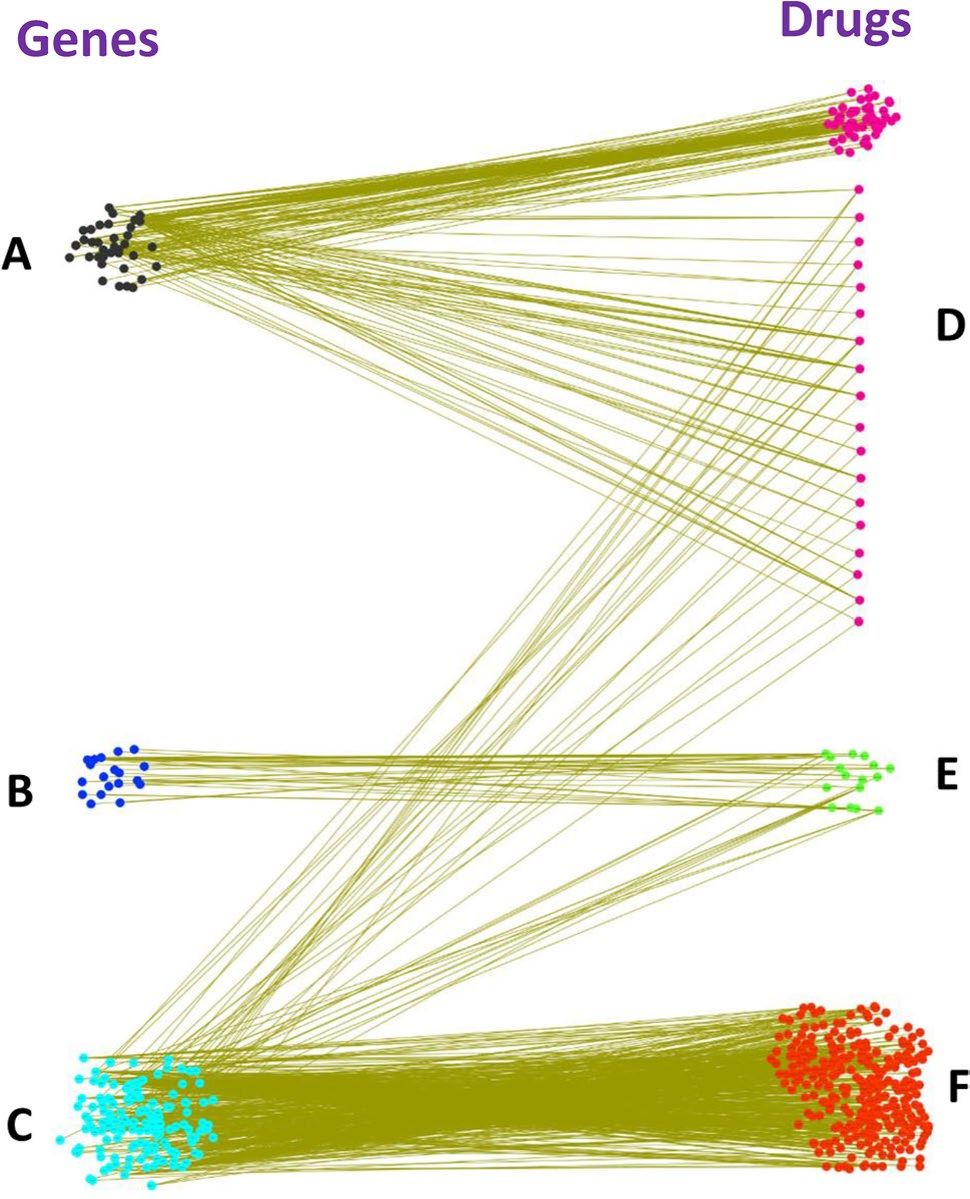
Robustness of gene ranking. (**A**–**C**) Gene ranking of total 202 targets of at least one of the 380 drugs (Supplementary Table S2). (**A**) Top 37 genes lie on the lower circled portion (mean FCG$<10^{-6}$) in Fig. 1. (**B**) 19 Genes lie in between the lower and upper circled portion (mean FCG lies between $10^{-6}$ to 0.99995) in Fig. 1. (**C**) Bottom 146 genes lie on the upper circled portion (mean FCG$> 0.99995$) in Fig. 1. (**D**) 73 drugs (mean FCG$<10^{-6}$) significantly reduce the growth rate of cancer models. (**E**) 16 drugs which gives mean FCG between $10^{-6}$ to 0.99995 in cancer models. (**F**) 291 drugs that show no effect (mean FCG $> 0.99995$) in the cancer models.

We used our gene knockout results of 202 target genes corresponding to those 380 drugs to test the significance of gene ranking. It was observed that 37 genes were present at the top position and 146 genes at the bottom in our gene ranking list (see Supplementary Table S2). The drugs corresponding to these 37 genes significantly reduced (mean FCG $<10^{-6}$) the growth rate of cancer models (Fig. 8). On the contrary, those drugs whose targets belong to the set of 146 genes, placed at the bottom in our gene ranking list, showed no effect in the cancer models.

## Discussion

Recently, GSMM gained a lot of attention in drug discovery. It has been used to study drugs related to cancer. Ghaffari et al.^47^ explored strategy for identifying anti-growth factors for the inhibition of cell growth using GSMM on 11 cell-lines and identified potential antimetabolites that could inhibit the growth or kill any of the cell-lines. They also checked the in-silico toxicity by employing GSMMs for 83 human healthy cell-types. The same methodology had been applied by Agren et al.^30^ in a different study to find potential drugs for hepatocellular carcinoma (HCC) by reconstructing and analysing personalized GSMMs for six HCC patients. Raškevičius et al.^6^ used GSMMs to predict the putative effects against cancer of those compounds which are structurally similar to human metabolite and also gave a concept of finding therapeutic windows through GSMMs. Turanil et al.^48^ introduced a drug repositioning based method via GSMMs to predict therapeutic agents for cancer treatment. They reconstructed prostate cancer-specific GSMM by combining personalized GSMMs (*n* > 450) and proteomics data. They used drug-perturbed gene expression data of three cell-lines (PC3, HL60, and MCF7) from the ConnectivityMap2 (CMap2)^49^ to reveal drug off-targets by predicting novel gene–drug interactions and evaluated in-silico cell viability. The present study aimed to develop a knockout strategies for identifying potential drug targets and the associated mechanisms using GSMM and gene expression data. This will help us to get novel drug targets as well as targets that might be used for drug repurposing. We used existing GSMMs of NCI-60 cell-lines^29^ to predict the anti-proliferative activity of single metabolic genes as targets against cancer and ranked them accordingly. We got 143 genes whose knockout reduced the cell growth across all the metabolic models of the NCI-60 panel. We also obtained a list of 1488 genes whose knockout does not show any effect on the growth rate of any cancer model. We searched for the underlying mechanism for such reduction in the growth rate of cancer cells by 143 genes and found that the biomass reduction score (BRS) of those genes were much higher than those of 1488 genes. The synergic effect in biomass reaction is much more for 143 gene list than 1488 gene list. It is observed that there are 12 biomass metabolites which are influenced by almost all the 143 genes but not by any of the 1488 genes for all the 60 cell-lines. Glycogen is the top-ranked among those 12 biomass metabolites. It is already reported that glycogen pathway is up-regulated in various cancers^50,51^ and funneled into glycolysis to promote cell growth, invasion and metastasis^52^. Likewise, glycogen is used by cancer cells to survive under nutrient starvation condition^53^. Considering its importance, inhibition of glycogen metabolism has become a new potential strategy for cancer treatment^50,54–56^.

We looked for potential targets from these 143 genes which showed a significant reduction in the growth rate following knockout. To be a potential target, these genes need to show the minimal knockout effect on the normal cells. We obtained 13 such targets from 143 genes whose knockout reduced the proliferation rate of cancer cells but were inactive across all the 9 normal cell models. One of the main features of the identified targets was that they were showing their effect in all the NCI-60 cell-lines. To experimentally validate the effect of these targets on multiple cell-lines, we chose SOAT1 and CYTB and showed that it’s inhibition reduces the growth rate in multiple cell-lines. Inhibition of SOAT1 is known to reduce growth rate in 5 cell-lines^42^ and additionally we have showed its effect on 4 more cell-lines. These cell-lines are taken from different tissues like blood peripheral, colon, adrenocortical gland, cervix, liver, kidney and brain. We have used mitotane to inhibit SOAT1, which is already known for the treatment of adrenocortical carcinoma and Cushing’s syndrome^57–59^. However, the inhibitor of CYTB, myxotyiazol, is not known as an anticancer drug, but we have seen its growth inhibiting effect on four cancer cell-lines. This could be a potential novel repurposed drug and need further evaluation. Moreover, literature survey showed that inhibition of SOAT1 and CYTB do not have any significant influence on the growth of the normal cells^60–62^. Literature also supports the validity of our other identified targets (given in Table 1). For example, atovaquone, a potent and selective mitochondrial inhibitor^63,64^, has been shown to reduce proliferation in cervical cancer cell-lines^65^, Du145 prostate cancer cells^66^ and MCF7-derived Cancer Stem-like CSCs^62^. Another identified target is UQCRB, whose inhibitor terpestacin blocks vascular endothelial growth factor (VEGF)-induced angiogenesis in endothelial cells and is proposed to be applied as a drug for human cancer^67,68^. Cytochrome c-1 (CYC1) is found to play an important role in breast cancer patients. Knocking down of CYC1 inhibits proliferation in human breast cancer cell-lines^69^. In another study, it was observed that silencing CYC1 by shRNA transfection also inhibits proliferation in human osteosarcoma (OS) cells^70^. Another identified gene target UQCRFS1 appears to be involved in the progression of gastric cancers and in the development of more aggressive phenotype of breast cancer^71,72^. Lentivirus-mediated knockdown of UQCRC2 suppresses cell growth and colony formation in RKO and HCT116 cells, result in cell cycle arrest and induce cell apoptosis in vitro in colorectal cancer (CRC)^73^. The knockdown of ACACA expression inhibits cell proliferation in prostate^74^ and breast^75^ cancer cell-lines.

We used Spearman rank-correlation method to compare our single-gene knockout ranking obtained from the GSMM with the experimental data. Most of the observed rank-correlations were very low and/or not significant. This was in agreement with other studies where single-gene knockout does not show the desired result^76–78^. On the other hand, when applied multiple gene knockout strategies, we obtained a higher and significant rank-correlations with the experimental results. It leads to the conclusion that multiple genes knockout show a better result than single-gene knockout, confirming the similar observations by other studies^77–81^. This might be because genes or proteins interact in a complex network, where alternative pathways always exist to carry the function^77,82^. Though the single knockout results did not give the desired correlations, the gene ranking obtained using this strategy seemed to be significant. It was noticed that a drug could only be active if it has at least one target belonging to the top rank. In case, none of the targets is from the top rank then that drug is observed to be ineffective.

The developed strategy to use gene knockout in GSMMs to identify drug targets has general applicability with any novel set of gene expression data associated with a tumour. The tumour-specific GSMM can be built from the gene expression data to go for the single or multiple gene knockout strategies. We proposed to use drug information for multiple gene knockout strategies that can also serve as drug repurposing technique. This method could be used to identify novel drug targets as well as targets that might be used by existing drugs on novel kinds of tumour. It is, therefore, possible to identify genes that are more relevant to specific cancer. The developed methodology could also be used to screen for common therapy. This strategy was used in the present study where we obtained 143 genes whose knockout were showing significant growth reduction across all cell-lines (see Supplementary Fig. S2)

Finally, we want to mention that the GSMM models use the metabolic flux values of reactions for prediction. It is known that these fluxes depend on protein’s post-translational state as well as corresponding metabolite levels^83^. More detailed data of these cell-lines (proteomics and metabolomics)^84–86^ would help to construct a more accurate model of cancer cell-lines and will certainly enhance the possibility of finding new targets.

## Materials and methods

### Predicting cancer cell growth by gene knockout

We used minimization of metabolic adjustment (MOMA) technique^33^ for observing gene knockout effect on the growth rates of 60 cancer cell-line models. To mimic the knockout condition of a gene, we changed the lower and upper bounds of flux values of the reactions associated with the gene to zero. In the case of simultaneous knockout of multiple genes, the bounds of all reactions of these participating genes were set to zero. To measure the growth rate, we used two models as input for MOMA: one representing the knockout condition and the other one representing the wild-type condition of a particular cell-line. The fractional cell growth (FCG) was then calculated by taking the ratio of growth rates in knockout condition to wild-type condition. COBRA toolbox was used from the MATLAB package^34^ for MOMA. Though there exists several genes associated with the same reaction with non-trivial *AND* and *OR* combinations, in order to keep our analysis simple, we followed the literatures^27,29,87,88^ and assumed zero flux for the reaction associated with the knockout gene.

### Gene symbol to Gene ID conversion

In GSMMs, the identity of genes is given in gene ID format and therefore the gene symbols need to be converted into gene IDs. For this purpose, we used the Uniprot database, which provides the mapping between gene IDs and gene symbols of the metabolic genes. Using this method, one can find out the metabolic genes from any given gene set having gene ID information. One can also extract genes from any given gene set, which are present in the cancer cell-line metabolic models, by taking intersection with the 1905 gene IDs present in the cancer models.

### Gene knockout effect on the production flux rate of metabolite

Flux balance analysis (FBA) was applied to get the flux values of each of the metabolic reactions present in the metabolic networks under the wild-type and gene knockout condition. Then, the production flux rate of a metabolite was obtained by summing up the flux values of the reactions in which it is produced. Finally, the ratio between the production flux rates under the two different conditions was calculated.

### Biomass reduction score (BRS)

Total 43 metabolites are used as the substrate in the biomass reaction, and we termed them as biomass metabolites (see Supplementary Table S1). These biomass metabolites are then arranged in the decreasing order of their coefficient values in the biomass reaction and scored them accordingly. For example, the biomass metabolite having the highest coefficient was scored 43 and the biomass metabolite with the lowest coefficient was scored 1. To capture the individual gene knockout effect on the biomass reaction, we first listed out the biomass metabolites whose production flux rates are reduced by more than twofolds. We then calculated the sum of the scores of these biomass metabolites and termed them as biomass reduction score (BRS).

### MTT assay

Different cancerous cell-lines were maintained at 37 $^{\circ }$C in a humidified incubator with 5% $\hbox {CO}_{2}$ supply. A549 (Human lung carcinoma), HCT116 (Human colorectal carcinoma), K562 (Human chronic myelogenous leukaemia) cells were grown in Dulbecco’s Modified Eagle Medium (DMEM) (Hyclone, West South logan, USA) and HL60 (Human promyeloblast) was grown in RPMI 1640 (Gibco) supplemented with 10% fetal bovine serum (FBS). The SOAT1 inhibitor mitotane and CYTB inhibitor myxothiazol were purchased from Sigma Aldrich (St. Louis, USA). They were dissolved in dimethyl sulfoxide (DMSO) (Sigma-Aldrich, MO, United States) and stored in $-20\;^{\circ }\hbox {C}$ as a stock concentration of 10 mM and 50 mM respectively. The effect of mitotane and myxothiazol on the proliferation and viability of all cell-lines was monitored using 3-(4,5-dimethylthiazol-2-yl)-2,5-diphenyltetrazolium bromide (MTT) assay. MTT powder was purchased from Merck, USA and was dissolved in PBS (5 mg/ml) prior to the assay. MTT assay is based on the principle of reduction of 3-(4,5-dimethylthiazol-2-yl)-2,5-diphenyltetrazolium bromide to purple coloured formazan by the mitochondria of metabolically active cells. Cells were seeded at a density of $2\times 10^4$ cells per well in 96 well plate for 24 h followed by addition of the drug at different concentrations. The MTT assay was performed after 48 h of incubation with the drug. Each experiment was performed three times with five replicates. Mean, standard deviation and standard error were determined using Graphpad prism.

### Extraction of experimental gene knockdown information from database

We used DEMETER database^44^ to obtain the experimental gene knockdown effect on cell growth. It contains a knockdown effect on the viability of cells from 501 cancer cell-lines using the shRNA library. The data is given in the form of log fold change in cell number due to gene knockdown. A gene with a negative log fold change value implies that there is a reduction in the cell growth upon knockdown of this gene^44^. The database contains the knockdown results of 17,098 genes on 501 cell-lines, out of which 30 cell-lines are present in NCI-60 cell-lines panel. In our cancer models, we got only 1444 genes from those 17,098 genes. So, we obtained the gene knockdown effect of 1444 metabolic genes on 30 cancer cell-lines.

### Permutation test

Permutation test was applied to get the *p*-value corresponding to each Spearman rank-correlation. In the first step, Spearman rank-correlation was calculated between the predicted data and the experimental data. Next, the predicted data were randomly permuted by using the *randperm* function in MATLAB. The function *randperm* gives a randomly permuted vector of the integers from 1 to *n* without repeating elements. In our case, *n* represents the number of data points. The elements of the randomly generated vector is used as index of the original data to generate the permuted data. Spearman rank-correlations were calculated between the permuted data and the experimental data. This process was repeated for 1000 times and the *p*-value was obtained by using the formula $(r+1)/1001$, where r is the number of cases for which random permuted set gave better rank-correlation than the non-permuted case.

To verify the usefulness of the permutation test we obtained the distribution of the rank-correlations for the 1000 randomly permuted set (see Supplementary Fig. S3 for three cell-lines). It was observed that they follow normal distribution.

### Finding drugs with inhibitory type nature from DrugBank database

In DrugBank database^45^, 6490 drugs (out of 8283) have information about its targets and the corresponding mechanism of action. These targets are given in the form of gene names. There are 1684 drugs which have gene ID information of at least one metabolic target (see method “Gene symbol to Gene ID conversion”). We then searched for the drugs that can decrease the activity of at least one metabolic target. For this purpose, we selected the following three mechanisms of actions: (i) inhibition, (ii) antagonist, (iii) inverse agonist. We found 410 drugs out of 1684 which decreased the activity of at least one metabolic gene. Out of these 410 drugs, only 380 drugs have at least one inhibitory type target on cancer cell-lines GSMMs.

### Link between DrugBank database and NCI-60 growth inhibition database

DrugBank database has DrugBank ID, whereas NCI-60 growth inhibition database^46^ has NSC ID. So, we first converted their IDs into a single ID for further analysis. In the DrugBank database, the conversion of drug ID to PubChem CID or CAS ID is provided and the conversion of CAS ID to NSC ID is given in the chemical data section of the NCI-60 growth inhibition database. We therefore used CAS ID information of the drugs to link these two datasets. There are 373 drugs, out of previously described 380 drugs, which have CAS ID information and out of them only 200 drugs have NSC IDs. However, all NSC IDs do not have information regarding GI50 value. So, we finally obtained 64 drugs with NSC IDs that have a GI50 score in negative log value.

### Finding active drugs using GI50 score

GI50 score is an important measure of drug activity. It quantifies the dosage of the drug required to inhibit the cell growth by $50\%$. In the NCI-60 growth inhibition database, the range of log value of GI50 score of the drugs is given between − 10 to − 1. Drugs with log GI50 score less than − 5 was considered to be active against cancer^89,90^.

## Supplementary information

Supplementary Information 1

Supplementary Information 2

Supplementary Information 3

Supplementary Information 4

## Data Availability

The data and the code are provided as Supplementary File S1, Supplementary Table S1 and Supplementary File S2.
