# Supplementary material for: Exploring gene knockout strategies to identify potential drug targets using genome-scale metabolic models
Source: Sci Rep. 2021 Jan 8;11:213. doi: 10.1038/s41598-020-80561-1 (PMC7794450; doi:10.1038/s41598-020-80561-1)
Supplement: Supplementary file 2 — Supplementary Information 2 [file 41598_2020_80561_MOESM2_ESM.zip › Exploring_gene_knockout_strategies_metabolic_models_Paul_et.al._Supplementary_File_S2/Gene_KO.pdf]

```
%This codes calculates the fractional cell growth (FCG) for each gene in
%all 60 cancer cell-line models. This code uses the Gurobi solver.

clc
clear
initCobraToolbox()
changeCobraSolver('gurobi5','QP')
vec=[];
for i=1:60
    % load each models one by one. "readCbModel" function reads the SBML
    % file and converts into a '.mat' file. This funcion can be found in
    % the COBRA Toolbox
    % NOTE: To get the models, see Yizhak, K. et al., 2014.
    % https://doi.org/10.7554/eLife.03641.001

    %Saving the original model before knockout
    origmodel=model;

    for j=1:length(origmodel.genes)
        model=origmodel;

        %Genaration of knockout model
        r=find(model.rxnGeneMat(:,j));
        for k=1:length(r)
            model.lb(r(k))=0;
            model.ub(r(k))=0;
        end

        % MOMA gives growth rate after knockout. Codes of MOMA is given in
        % the COBRA Toolbox
        [c, d]=MOMA(origmodel,model);
        vec(j,i)=c.f/d.f;
   end
end
% save vec vec;

% Run to generate Figure 1
% load(model and vec)
figure;plot(sort(mean(vec,2)),'.-');

mn=mean(vec,2); % Average FCG value
sd=std(vec,0,2); % Standard deviation
% 143 genes which show very low growth rate after knockout cancer models.
a=find(mn<1.0000e-06);
model.genes(a);
% 1488 genes which show negligible effect on the growth rate.
b=find(mn>=0.99999);
model.genes(b);
```
