# Supplementary material for: Exploring gene knockout strategies to identify potential drug targets using genome-scale metabolic models
Source: Sci Rep. 2021 Jan 8;11:213. doi: 10.1038/s41598-020-80561-1 (PMC7794450; doi:10.1038/s41598-020-80561-1)
Supplement: Supplementary file 2 — Supplementary Information 2 [file 41598_2020_80561_MOESM2_ESM.zip › Exploring_gene_knockout_strategies_metabolic_models_Paul_et.al._Supplementary_File_S2/Gene_KO_validation.pdf]

```
%This codes compares the predicted gene knockout data with experimentally
%observed gene knockdown data.
%NOTE: To get the experimental data, see DEMETER database,
%Lamb, J. et al., 2006.
%DOI: 10.1126/science.1132939
%or visit https://depmap.org/portal/download/((Achilles 2.20.2)

%load Model predicted data and renamed as "val_model"
%load data downloaded from DEMETER database and renamed as "val_demeter"
%NOTE: The size of these two matrix must be same

KO_ac_S=[];
for i=1:size(val_model,2)
    ans1=[reshape(val_model(:,i),[],1),reshape(val_demeter(:,i),[],1)];
    isnan(ans1(:,2));
    ans1=ans1(find(ans==0),:);
    [R,P] = corr(ans1(:,1),ans1(:,2),'Type','Spearman', 'Tail','right');
    v=[];

    % Permutation test
    rng(1)
    for j=1:1000
        ans2=[ans1(randperm(length(ans1)),1),ans1(:,2)];
        [R1,P1] = corr(ans2(:,1),ans2(:,2),'Type','Spearman',
'Tail','right');
        v(j,1)=R1;
    end
    KO_ac_S(i,1)=R;
    KO_ac_S(i,2)=(length(find(v>R))+1)/1001;
end
% save KO_ac_S KO_ac_S

figure;plot(KO_ac_S(:,1),KO_ac_S(:,2),'.')
% median(KO_ac_S(:,1)); % median Spearman rank-correlation
signrank(KO_ac_S(:,1)); % Wilcoxon's signed rank p-value
```
