# Supplementary material for: Exploring gene knockout strategies to identify potential drug targets using genome-scale metabolic models
Source: Sci Rep. 2021 Jan 8;11:213. doi: 10.1038/s41598-020-80561-1 (PMC7794450; doi:10.1038/s41598-020-80561-1)
Supplement: Supplementary file 2 — Supplementary Information 2 [file 41598_2020_80561_MOESM2_ESM.zip › Exploring_gene_knockout_strategies_metabolic_models_Paul_et.al._Supplementary_File_S2/Mechanistic.pdf]

```
% This codes gives the Mechanistic insight into the genes giving a low growth
rate after knockout

%% Parsimonious enzyme usage FBA (pFBA)

clc
clear
initCobraToolbox()
changeCobraSolver('gurobi5','QP')

p_FBA={};
for i=1:60
    % load each models one by one. "readCbModel" function reads the SBML
    % file and converts into .mat file. This funcion can be found in
    % the COBRA Toolbox
    % NOTE: To get the models, see Yizhak, K. et al., 2014.
    % https://doi.org/10.7554/eLife.03641.001

    % Codes of pFBA can be found in the COBRA Toolbox
    [GeneClasses RxnClasses modelIrrevFM] = pFBA(model, 'geneoption',0,
'tol',1e-7);
    p_FBA{1,i}=GeneClasses.pFBAEssential;
    p_FBA{2,i}=GeneClasses.pFBAoptima;
    p_FBA{3,i}=GeneClasses.ELEGenes;
    p_FBA{4,i}=GeneClasses.MLEGenes;
    p_FBA{5,i}=GeneClasses.ZeroFluxGenes;
    p_FBA{6,i}=GeneClasses.Blockedgenes;
end
% save p_FBA p_FBA


%% Biomass metabolites

% load any cancer cell-lines model
ans1=find(model.S(:,find(model.c))<0);
[a,b]=sort(full(model.S(ans1,find(model.c))));
meta_bio=ans1(b);
% save meta_bio meta_bio


%% Calculation of biomass reduction score (BRS) for each gene

clc
clear
initCobraToolbox()
changeCobraSolver('gurobi5','QP')
load('meta_bio.mat')
BRS=[];meta_prod={};
for i=1:60
    % load each models one by one. "readCbModel" function reads the SBML
    % file and converts into .mat file. This funcion can be found in
    % the COBRA Toolbox
    % NOTE: To get the models, see Yizhak, K. et al., 2014.
    % https://doi.org/10.7554/eLife.03641.001
```

```
    %Saving the original model before knockout
    origmodel=model;

    % FBA of original model
    wt= optimizeCbModel(origmodel);

    A=zeros(length(origmodel.genes),length(meta_bio));
    for j=1:length(origmodel.genes)
        i
        j
        model=origmodel;

        %Genaration of knockout model
        r=find(model.rxnGeneMat(:,j));
        for k1=1:length(r)
            model.lb(r(k1))=0;
            model.ub(r(k1))=0;
        end

        % FBA of knockout model
        kn = optimizeCbModel(model);

        % Calculation of production flux of biomass metabolites
        wt_bio=zeros(1,length(meta_bio)); % Wild-type model
        kn_bio=zeros(1,length(meta_bio)); % Knockout model
        for k2=1:length(meta_bio)
            ans1=find(model.S(meta_bio(k2),:));
            ans2_1=kn.x(ans1);
            ans2_2=wt.x(ans1);
            ans3=full(model.S(meta_bio(k2),ans1));
            ans3=ans3';
            ans4_1=ans2_1.*ans3;
            ans4_2=ans2_2.*ans3;
            kn_bio(1,k2)=sum(ans4_1(find(ans4_1>0)));
            wt_bio(1,k2)=sum(ans4_2(find(ans4_2>0)));
        end

        % metabolites whose production fluxes are reduced by 2-fold
        % in knockout condition
        ans5=find(kn_bio./wt_bio<0.5);
        A(j,ans5)=1;

        % BRS score for each gene
        BRS(j,i)=44*length(ans5)-sum(ans5);
    end
    meta_prod{1,i}=A;
end

% save BRS BRS
% save meta_prod meta_prod
```
