# Supplementary material for: Exploring gene knockout strategies to identify potential drug targets using genome-scale metabolic models
Source: Sci Rep. 2021 Jan 8;11:213. doi: 10.1038/s41598-020-80561-1 (PMC7794450; doi:10.1038/s41598-020-80561-1)
Supplement: Supplementary file 2 — Supplementary Information 2 [file 41598_2020_80561_MOESM2_ESM.zip › Exploring_gene_knockout_strategies_metabolic_models_Paul_et.al._Supplementary_File_S2/README.pdf]

README:

Have to install the COBRA Toolbox and the Gurobi software to run the codes. These two are freely available.

The whole computational procedure should be run as follows (Each of the scripts below is fully documentized):

1. Gene_KO- Calculating the fractional cell growth (FCG) for each gene in all 60 cancer cell-line models.

2. Mechanistic - Giving mechanistic insight into the genes giving a low growth rate after knockout.

3. Gene_KO_validation - Comparing the predicted gene knockout data with experimentally observed gene knockdown data.
