# Supplementary material for: Exploring gene knockout strategies to identify potential drug targets using genome-scale metabolic models
Source: Sci Rep. 2021 Jan 8;11:213. doi: 10.1038/s41598-020-80561-1 (PMC7794450; doi:10.1038/s41598-020-80561-1)
Supplement: Supplementary file 3 — Supplementary Information 3 [file 41598_2020_80561_MOESM3_ESM.pdf]

Exploring gene knockout strategies to identify potential drug targets using genome-scale metabolic models

Abhijit Paul[1], Rajat Anand[1], Sonali Porey Karmakar[1], Surender Rawat[1], Nandadulal Bairagi[2], and Samrat Chatterjee[1,*]

[1]Complex Analysis Group, Translational Health Science and Technology Institute, NCR Biotech Science Cluster, 3rd milestone, Faridabad-Gurgaon Expressway, Faridabad-121001, India
[2]Centre for Mathematical Biology and Ecology, Department of Mathematics, Jadavpur University, Kolkata-700032, India
[*]samrat.chatterjee@thsti.res.in

Supplementary Figures:

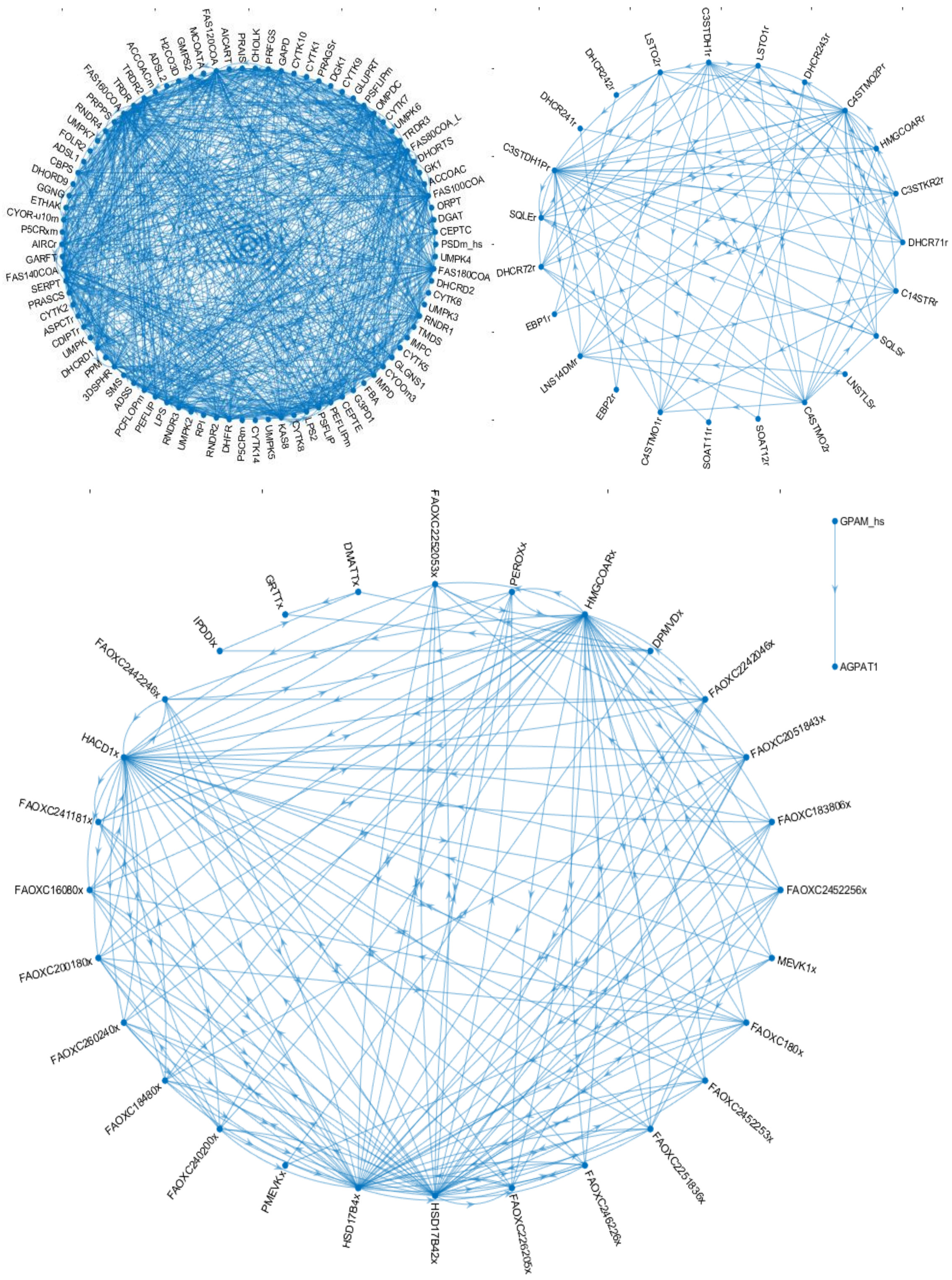

Figure S1: **Flux coupled reactions corresponding to the 143 growth-reducing genes.** In total, 176 reactions are associated to the 143 gene set.Among these 176 reactions, 143 reactions formed group of coupled reactions which is presented here. There are only one genes out of 143, whose any associated reactions are not present in these coupled reaction set.

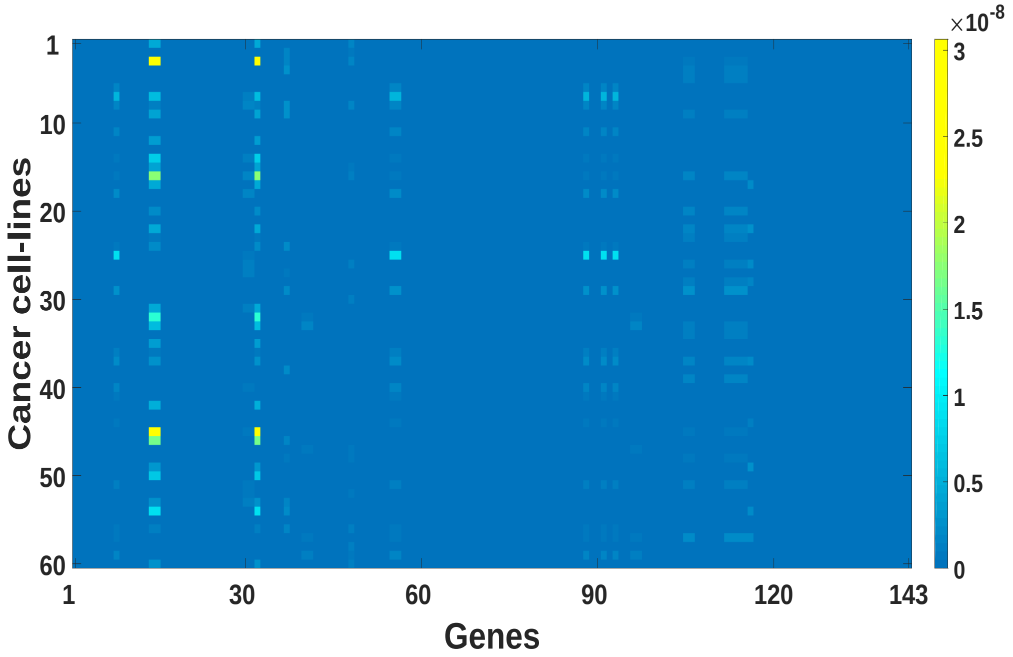

Figure S2: **Gene knockout simulation result for 143 genes across 60 cancer cell-line models.** Here the colour bar represents the fractional cell growth (FCG), the ratio of growth rates in knockout condition to wild-type condition. Here, we observed very low FCG value across all the 60 cancer models.

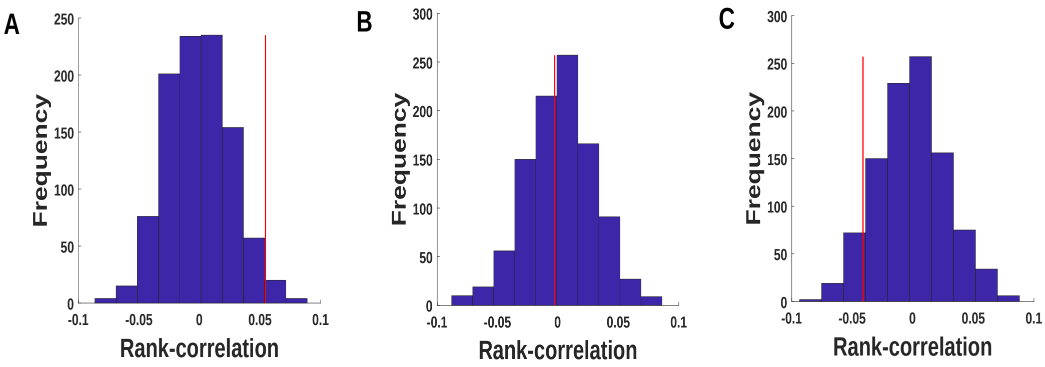

Figure S3: **Distribution of Rank-correlation for the 1000 randomly permuted set.** The red line represents the rank-correlation for the non-permuted case. We presented here three cases, where the rank-correlation for the non-permuted cases are: (A) Positive, (B) Almost zero and (C) Negative. "Anderson-Darling test" was applied to check the normality. This give a test decision for the null hypothesis that the vector is coming from a population with a normal distribution. In our case we got zero value which indicates that the test fails to reject the null hypothesis at 5% significance level.
