# Supplementary material for: Exploring gene knockout strategies to identify potential drug targets using genome-scale metabolic models
Source: Sci Rep. 2021 Jan 8;11:213. doi: 10.1038/s41598-020-80561-1 (PMC7794450; doi:10.1038/s41598-020-80561-1)
Supplement: Supplementary file 4 — Supplementary Information 4 [file 41598_2020_80561_MOESM4_ESM.docx]

**Supplementary Information**

**Exploring gene knockout strategies to identify potential drug targets using genome-scale metabolic models**

Abhijit Paul^1^, Rajat Anand^1^, Sonali Porey Karmakar^1^, Surender Rawat^1^, Nandadulal Bairagi^2^, Samrat Chatterjee^1^*

^1^Complex Analysis Group, Translational Health Science and Technology Institute, NCR Biotech Science Cluster, 3rd milestone, Faridabad-Gurgaon Expressway, Faridabad-121001, India

^2^Centre for Mathematical Biology and Ecology, Department of Mathematics, Jadavpur University, Kolkata-700032, India

*Corresponding author: samrat.chatterjee@thsti.res.in

**Supplementary Table S1:** The list of metabolites that are used as substrate in the Biomass reaction. We listed them accordingly to their coefficients given in the biomass reaction. Negative sign represents the consumption of this metabolites in biomass reaction.

| **Metabolites Names** | **Metabolites Symbol** | **Coefficient in Biomass reaction** | **Rank** | **Score** |
| --- | --- | --- | --- | --- |
| ATP | atp[c] | -43.153 | 1 | 43 |
| H2O | h2o[c] | -43.153 | 2 | 42 |
| L-Aspartate | asp-L[c] | -2.452366 | 3 | 41 |
| glycogen, structure 1 (glycogenin-11[1,4-Glc]) | glygn1[c] | -0.939899 | 4 | 40 |
| L-Glutamine | gln-L[c] | -0.676289 | 5 | 39 |
| L-Glutamate | glu-L[c] | -0.594216 | 6 | 38 |
| Glycine | gly[c] | -0.492442 | 7 | 37 |
| L-Alanine | ala-L[c] | -0.420217 | 8 | 36 |
| L-Serine | ser-L[c] | -0.131318 | 9 | 35 |
| L-Histidine | his-L[c] | -0.10177 | 10 | 34 |
| triacylglycerol (homo sapiens) | tag_hs[c] | -0.097285 | 11 | 33 |
| L-Threonine | thr-L[c] | -0.072226 | 12 | 32 |
| phosphatidylethanolamine (homo sapiens) | pe_hs[c] | -0.07098 | 13 | 31 |
| L-Proline | pro-L[c] | -0.063691 | 14 | 30 |
| Phosphatidylcholine (homo sapiens) | pchol_hs[c] | -0.060784 | 15 | 29 |
| phosphatidic acid (homo sapiens) | pa_hs[c] | -0.058105 | 16 | 28 |
| L-Asparagine | asn-L[c] | -0.042678 | 17 | 27 |
| L-Valine | val-L[c] | -0.042678 | 18 | 26 |
| L-Leucine | leu-L[c] | -0.039396 | 19 | 25 |
| GMP | gmp[c] | -0.037088 | 20 | 24 |
| L-Lysine | lys-L[c] | -0.032829 | 21 | 23 |
| CMP | cmp[c] | -0.032723 | 22 | 22 |
| Cholesterol | chsterol[c] | -0.029279 | 23 | 21 |
| cholesterol ester | xolest_hs[c] | -0.023261 | 24 | 20 |
| phosphatidylserine (homo sapiens) | ps_hs[c] | -0.020854 | 25 | 19 |
| L-Tyrosine | tyr-L[c] | -0.019696 | 26 | 18 |
| AMP | amp[c] | -0.019636 | 27 | 17 |
| UMP | ump[c] | -0.019636 | 28 | 16 |
| monoacylglycerol 2 (homo sapiens) | mag_hs[c] | -0.013664 | 29 | 15 |
| L-Isoleucine | ile-L[c] | -0.013133 | 30 | 14 |
| L-Phenylalanine | phe-L[c] | -0.013133 | 31 | 13 |
| diacylglycerol (homo sapiens) | dag_hs[c] | -0.01114 | 32 | 12 |
| phosphatidylinositol (homo sapiens) | pail_hs[c] | -0.010963 | 33 | 11 |
| sphingomyelin (homo sapiens) | sphmyln_hs[c] | -0.008545 | 34 | 10 |
| dAMP | damp[c] | -0.00805 | 35 | 09 |
| dTMP | dtmp[c] | -0.00805 | 36 | 08 |
| L-Methionine | met-L[c] | -0.006567 | 37 | 07 |
| dCMP | dcmp[c] | -0.005367 | 38 | 06 |
| dGMP | dgmp[c] | -0.005367 | 39 | 05 |
| lysophosphatidylcholine (homo sapiens) | lpchol_hs[c] | -0.004474 | 40 | 04 |
| L-Cysteine | cys-L[c] | -0.004269 | 41 | 03 |
| L-Arginine | arg-L[c] | -0.003282 | 42 | 02 |
| L-Tryptophan | trp-L[c] | -0.003282 | 43 | 01 |

**Supplementary Table S2:** Gene ranking list of the total 202 genes which are the targets of at least one of the 380 DrugBank drugs. Top 37 genes lie on the lower circled portion in Fig. 1 (i.e. mean fractional cell growth (FCG) < 10^-6^) and bottom 146 genes lie on the upper circled portion in Fig. 1 (i.e. mean fractional cell growth (FCG) > 0.99995).

| **Rank** | **Gene ID** | **Mean fractional growth reduction across 60 cancer models** | **Standard deviation** |
| --- | --- | --- | --- |
| 1 | 1723.1 | 0 | 1.21E-09 |
| 2 | 4519.1 | 0 | 1.05E-10 |
| 3 | 23632.1 | 0 | 4.87E-13 |
| 4 | 377677.1 | 0 | 4.87E-13 |
| 5 | 759.1 | 0 | 4.87E-13 |
| 6 | 760.1 | 0 | 4.87E-13 |
| 7 | 761.1 | 0 | 4.87E-13 |
| 8 | 762.1 | 0 | 4.87E-13 |
| 9 | 766.1 | 0 | 4.87E-13 |
| 10 | 766.2 | 0 | 4.87E-13 |
| 11 | 768.1 | 0 | 4.87E-13 |
| 12 | 771.1 | 0 | 4.87E-13 |
| 13 | 771.2 | 0 | 4.87E-13 |
| 14 | 10423.1 | 0 | 0 |
| 15 | 10423.2 | 0 | 0 |
| 16 | 1595.1 | 0 | 0 |
| 17 | 2224.1 | 0 | 0 |
| 18 | 3156.1 | 0 | 0 |
| 19 | 4023.1 | 0 | 0 |
| 20 | 51727.1 | 0 | 0 |
| 21 | 6646.1 | 0 | 0 |
| 22 | 6713.1 | 0 | 0 |
| 23 | 2194.1 | 2.04E-14 | 2.57E-13 |
| 24 | 3614.1 | 1.62E-12 | 3.25E-11 |
| 25 | 3614.2 | 1.62E-12 | 3.25E-11 |
| 26 | 3615.1 | 1.62E-12 | 3.25E-11 |
| 27 | 7298.1 | 2.04E-10 | 1.05E-09 |
| 28 | 6240.1 | 3.13E-10 | 1.49E-09 |
| 29 | 6241.1 | 3.13E-10 | 1.49E-09 |
| 30 | 7296.1 | 3.13E-10 | 1.49E-09 |
| 31 | 7296.2 | 3.13E-10 | 1.49E-09 |
| 32 | 7296.3 | 3.13E-10 | 1.49E-09 |
| 33 | 7296.4 | 3.13E-10 | 1.49E-09 |
| 34 | 1719.1 | 3.36E-10 | 1.06E-09 |
| 35 | 2618.1 | 3.39E-10 | 1.68E-09 |
| 36 | 2618.2 | 3.39E-10 | 1.68E-09 |
| 37 | 471.1 | 3.39E-10 | 1.68E-09 |
| 38 | 2746.1 | 0.01699 | 0.091792 |
| 39 | 6470.1 | 0.054665 | 0.142623 |
| 40 | 6470.2 | 0.054665 | 0.142623 |
| 41 | 6392.1 | 0.134699 | 0.187822 |
| 42 | 4967.1 | 0.27278 | 0.179301 |
| 43 | 4967.2 | 0.27278 | 0.179301 |
| 44 | 38.1 | 0.717785 | 0.068667 |
| 45 | 124.1 | 0.871742 | 0.215248 |
| 46 | 125.1 | 0.871742 | 0.215248 |
| 47 | 126.1 | 0.871742 | 0.215248 |
| 48 | 18.1 | 0.871932 | 0.088614 |
| 49 | 18.2 | 0.871932 | 0.088614 |
| 50 | 3242.1 | 0.907608 | 0.032661 |
| 51 | 5053.1 | 0.952368 | 0.017447 |
| 52 | 36.1 | 0.953709 | 0.006882 |
| 53 | 4942.1 | 0.958361 | 0.005198 |
| 54 | 6523.1 | 0.961313 | 0.004169 |
| 55 | 2875.1 | 0.991049 | 0.023081 |
| 56 | 84706.1 | 0.991049 | 0.023081 |
| 57 | 6524.1 | 0.999999 | 2.04E-06 |
| 58 | 100.1 | 0.999999 | 1.98E-06 |
| 59 | 5334.1 | 0.999999 | 1.95E-06 |
| 60 | 1621.1 | 0.999999 | 1.92E-06 |
| 61 | 6559.1 | 0.999999 | 1.94E-06 |
| 62 | 6715.1 | 0.999999 | 1.87E-06 |
| 63 | 6716.1 | 0.999999 | 1.87E-06 |
| 64 | 231.1 | 0.999999 | 1.82E-06 |
| 65 | 2643.1 | 0.999999 | 1.79E-06 |
| 66 | 1588.1 | 0.999999 | 2.07E-06 |
| 67 | 1588.2 | 0.999999 | 2.07E-06 |
| 68 | 1645.1 | 0.999999 | 1.77E-06 |
| 69 | 3283.1 | 0.999999 | 1.77E-06 |
| 70 | 8398.1 | 0.999999 | 1.70E-06 |
| 71 | 8398.2 | 0.999999 | 1.70E-06 |
| 72 | 1374.1 | 0.999999 | 1.84E-06 |
| 73 | 1376.1 | 0.999999 | 1.84E-06 |
| 74 | 4842.1 | 0.999999 | 1.75E-06 |
| 75 | 4843.1 | 0.999999 | 1.75E-06 |
| 76 | 4843.2 | 0.999999 | 1.75E-06 |
| 77 | 4846.1 | 0.999999 | 1.75E-06 |
| 78 | 495.1 | 0.999999 | 1.70E-06 |
| 79 | 7173.1 | 0.999999 | 1.81E-06 |
| 80 | 7173.2 | 0.999999 | 1.81E-06 |
| 81 | 7173.3 | 0.999999 | 1.81E-06 |
| 82 | 7173.4 | 0.999999 | 1.81E-06 |
| 83 | 7173.5 | 0.999999 | 1.81E-06 |
| 84 | 291.1 | 0.999999 | 1.73E-06 |
| 85 | 292.1 | 0.999999 | 1.73E-06 |
| 86 | 293.1 | 0.999999 | 1.73E-06 |
| 87 | 3284.1 | 0.999999 | 1.61E-06 |
| 88 | 7498.1 | 0.999999 | 1.57E-06 |
| 89 | 26.1 | 0.999999 | 1.74E-06 |
| 90 | 4758.1 | 0.999999 | 1.64E-06 |
| 91 | 8639.1 | 0.999999 | 1.74E-06 |
| 92 | 2936.1 | 0.999999 | 1.65E-06 |
| 93 | 5320.1 | 0.999999 | 1.59E-06 |
| 94 | 2595.1 | 0.999999 | 1.77E-06 |
| 95 | 1312.1 | 0.999999 | 1.74E-06 |
| 96 | 1312.2 | 0.999999 | 1.74E-06 |
| 97 | 1584.1 | 0.999999 | 1.72E-06 |
| 98 | 1586.1 | 0.999999 | 1.72E-06 |
| 99 | 523.1 | 0.999999 | 1.67E-06 |
| 100 | 526.1 | 0.999999 | 1.67E-06 |
| 101 | 2739.1 | 0.999999 | 1.74E-06 |
| 102 | 5319.1 | 0.999999 | 1.66E-06 |
| 103 | 6570.1 | 0.999999 | 1.56E-06 |
| 104 | 6571.1 | 0.999999 | 1.56E-06 |
| 105 | 4128.1 | 0.999999 | 1.54E-06 |
| 106 | 19.1 | 0.999999 | 1.49E-06 |
| 107 | 10846.1 | 0.999999 | 1.57E-06 |
| 108 | 50940.1 | 0.999999 | 1.57E-06 |
| 109 | 5136.1 | 0.999999 | 1.57E-06 |
| 110 | 5136.2 | 0.999999 | 1.57E-06 |
| 111 | 5138.1 | 0.999999 | 1.57E-06 |
| 112 | 5139.1 | 0.999999 | 1.57E-06 |
| 113 | 5153.1 | 0.999999 | 1.57E-06 |
| 114 | 1644.1 | 0.999999 | 1.54E-06 |
| 115 | 6529.1 | 0.999999 | 1.50E-06 |
| 116 | 6538.1 | 0.999999 | 1.50E-06 |
| 117 | 6540.1 | 0.999999 | 1.50E-06 |
| 118 | 4953.1 | 0.999999 | 1.59E-06 |
| 119 | 5264.1 | 0.999999 | 1.59E-06 |
| 120 | 3251.1 | 0.999999 | 1.60E-06 |
| 121 | 57468.1 | 0.999999 | 1.60E-06 |
| 122 | 6783.1 | 0.999999 | 1.51E-06 |
| 123 | 5321.1 | 0.999999 | 1.49E-06 |
| 124 | 7357.1 | 0.999999 | 1.56E-06 |
| 125 | 8647.1 | 0.999999 | 1.67E-06 |
| 126 | 1503.1 | 0.999999 | 1.47E-06 |
| 127 | 2356.1 | 0.999999 | 1.51E-06 |
| 128 | 6539.1 | 0.999999 | 1.46E-06 |
| 129 | 4363.1 | 0.999999 | 1.53E-06 |
| 130 | 6916.1 | 0.999999 | 1.28E-06 |
| 131 | 6530.1 | 0.999999 | 1.47E-06 |
| 132 | 6531.1 | 0.999999 | 1.47E-06 |
| 133 | 5167.1 | 0.999999 | 1.41E-06 |
| 134 | 3176.1 | 0.999999 | 1.45E-06 |
| 135 | 2030.1 | 0.999999 | 1.58E-06 |
| 136 | 4535.1 | 0.999999 | 1.50E-06 |
| 137 | 4718.1 | 0.999999 | 1.50E-06 |
| 138 | 11238.1 | 0.999999 | 1.35E-06 |
| 139 | 763.1 | 0.999999 | 1.35E-06 |
| 140 | 2182.1 | 0.999999 | 1.51E-06 |
| 141 | 8972.1 | 1 | 1.29E-06 |
| 142 | 290.1 | 1 | 1.38E-06 |
| 143 | 5742.1 | 1 | 1.45E-06 |
| 144 | 5743.1 | 1 | 1.45E-06 |
| 145 | 121278.1 | 1 | 1.39E-06 |
| 146 | 7166.1 | 1 | 1.39E-06 |
| 147 | 6560.1 | 1 | 1.39E-06 |
| 148 | 11309.1 | 1 | 1.43E-06 |
| 149 | 6532.1 | 1 | 1.30E-06 |
| 150 | 30.1 | 1 | 1.31E-06 |
| 151 | 4129.1 | 1 | 1.42E-06 |
| 152 | 4353.1 | 1 | 1.27E-06 |
| 153 | 23657.1 | 1 | 1.21E-06 |
| 154 | 6476.1 | 1 | 1.25E-06 |
| 155 | 1583.1 | 1 | 1.25E-06 |
| 156 | 1786.1 | 1 | 1.25E-06 |
| 157 | 251.1 | 1 | 1.25E-06 |
| 158 | 2548.1 | 1 | 1.25E-06 |
| 159 | 279.1 | 1 | 1.25E-06 |
| 160 | 4759.1 | 1 | 1.25E-06 |
| 161 | 4907.1 | 1 | 1.25E-06 |
| 162 | 5033.1 | 1 | 1.25E-06 |
| 163 | 5406.1 | 1 | 1.25E-06 |
| 164 | 5740.1 | 1 | 1.25E-06 |
| 165 | 8513.1 | 1 | 1.25E-06 |
| 166 | 9453.1 | 1 | 1.25E-06 |
| 167 | 847.1 | 1 | 1.45E-06 |
| 168 | 7915.1 | 1 | 1.22E-06 |
| 169 | 7915.2 | 1 | 1.22E-06 |
| 170 | 5148.1 | 1 | 1.13E-06 |
| 171 | 5149.1 | 1 | 1.13E-06 |
| 172 | 8654.1 | 1 | 1.13E-06 |
| 173 | 8654.2 | 1 | 1.13E-06 |
| 174 | 8654.3 | 1 | 1.13E-06 |
| 175 | 6718.1 | 1 | 1.31E-06 |
| 176 | 476.1 | 1 | 1.21E-06 |
| 177 | 240.1 | 1 | 1.24E-06 |
| 178 | 27115.1 | 1 | 1.21E-06 |
| 179 | 5141.1 | 1 | 1.21E-06 |
| 180 | 5142.1 | 1 | 1.21E-06 |
| 181 | 5143.1 | 1 | 1.21E-06 |
| 182 | 5144.1 | 1 | 1.21E-06 |
| 183 | 5150.1 | 1 | 1.21E-06 |
| 184 | 5150.2 | 1 | 1.21E-06 |
| 185 | 3612.1 | 1 | 1.17E-06 |
| 186 | 3613.1 | 1 | 1.17E-06 |
| 187 | 1585.1 | 1 | 1.17E-06 |
| 188 | 2182.2 | 1 | 1.08E-06 |
| 189 | 5226.1 | 1 | 1.21E-06 |
| 190 | 217.1 | 1 | 1.24E-06 |
| 191 | 6557.1 | 1 | 1.06E-06 |
| 192 | 6558.1 | 1 | 1.06E-06 |
| 193 | 3290.1 | 1 | 9.67E-07 |
| 194 | 3290.2 | 1 | 9.67E-07 |
| 195 | 3291.1 | 1 | 9.67E-07 |
| 196 | 6546.1 | 1 | 1.06E-06 |
| 197 | 43.1 | 1 | 9.89E-07 |
| 198 | 43.2 | 1 | 9.89E-07 |
| 199 | 2977.1 | 1 | 8.43E-07 |
| 200 | 7299.1 | 1 | 7.68E-07 |
| 201 | 8288.1 | 1 | 6.06E-07 |
| 202 | 6548.1 | 1 | 6.29E-07 |
